# Inhibitory Effect of Japanese Traditional Kampo Formula Frequently Prescribed in Gynecological Clinics on CYP3A4

**DOI:** 10.1155/2018/4259603

**Published:** 2018-10-01

**Authors:** Hao Ni, Takashi Matsumoto, Junko Watanabe, Toshiaki Makino

**Affiliations:** ^1^Department of Pharmacognosy, Graduate School of Pharmaceutical Sciences, Nagoya City University, 3-1 Tanabe-dori, Mizuho-ku, Nagoya 467-8603, Japan; ^2^Tsumura Kampo Research Laboratories, Kampo Research & Development Division, Tsumura & Co., 3586 Yoshiwara, Ami-machi, Inashiki-gun, Ibaraki 300-1192, Japan

## Abstract

Recently, the use of herbal medicines has become popular, and information on drug interactions between herbal medicines and chemical drugs is needed in clinics. In Japan, the number of patients taking Japanese traditional Kampo medicines has been increasing, and the proper drug information about herb-drug interaction is highly demanded. The most established herb-drug interaction is the case of grapefruit juice (GFJ)* via* the inhibition on CYP3A4 expressed in the small intestine. In the present study, we compared the inhibitory titer on CYP3A4 between the target Kampo products and GFJ used as positive control. We evaluated the inhibitory effects of GFJ and three extracts of Kampo formulas frequently used in gynecological clinics on CYP3A4* in vitro* and calculated the related titer of one-time dosage of Kampo formulas to GFJ in order to predict its effect on clinics. Although the extracts of these three Kampo formulas and the most of crude drug components in the formulas exhibited the inhibitory effects on CYP3A4 in some levels, the possibilities of tokishakuyakusan and keishibukuryogan to cause drug interaction can be quite low; however, it is possible that the excessive dosage of kamishoyosan may cause drug interaction with the substrate of CYP3A4 in clinics.

## 1. Introduction

As the integrated medicine using both modern Western medicine and traditional medicines is getting popular, the possibilities of the interaction between them have been described [1–4]. Especially in Japan, the number of patients taking Japanese traditional Kampo medicines has been increasing, and drug information about modern medicine and Kampo medicine is highly required. The most famous drug interaction between herbal and chemical drugs is the case of grapefruit juice (GFJ) [5]. Furanocoumarins such as 6,7′-dihydroxybergamottin (DHBG) contained in GFJ inhibit CYP3A4 expressed in the small intestine, which results in the augmentation of the blood concentration of some drugs and their activities in the body [6]. Some Kampo medicines containing crude drugs derived from Umbelliferae plants containing furanocoumarins have a potent inhibitory effect on CYP3A4* in vitro*; however, these medicines did not cause drug interaction in rats [7, 8]. In previous studies, excessive dosage of Kampo medicines may inhibit or induce CYP3A4 in animal experiments or in* in vitro* study [9–12]; it is predicted that their effects are too week not to cause drug interaction in clinic [13]. Since the results of CYP inhibitions in* in vitro* experiments tended to exhibit excessive high potency, only a few show interactions large enough to be clinically important [14]. Indeed, the clinical studies to evaluate drug interaction between Kampo and modern medicines must exhibit the best drug information; however, it is very limited to conduct clinical studies by cost and ethical problems.

In order to provide correct drug information about herb-drug interaction* via* CYP3A4 inhibition to physicians and pharmacists, we consider the comparison of the inhibitory titer of CYP3A4 between the target herbal products and GFJ, since GFJ is the only natural substance to inhibit CYP3A4 in clinical studies and used as positive control in many clinical studies [14]. Kampo medicines have been used as the ethical or OTC formulations in Japan, and the ingredients, methods of preparation, and dosages are approved by Japanese Medicine Regulatory Agency [15]. And in Japan, the most users of traditional Kampo medicine are the physicians belonging to gynecologics [16]. In this study, we evaluated the inhibitory effects of GFJ and three Kampo medicines frequently used in gynecology clinics on CYP3A4 and calculated the related titer of one-time dosage of Kampo medicine to GFJ in order to predict its effect on clinics.

## 2. Material and Methods

### 2.1. Crude Drugs and Kampo Formulation

Tokishakuyakusan (Dangguishaoyaosan) (daily dose for human) consists of 4.0 g of the root of* Paeonia lactiflora* (Peony Root), 4.0 g of the rhizome of* Atractylodes lancea* (Atractylodes Lancea Rhizome), 4.0 g of the tuber of* Alisma orientale* (Alisma Tuber), 4.0 g of the sclerotium of* Wolfiporia cocos* (Poria Sclerotium), 3.0 g of the rhizome of* Cnidium officinale* (Cnidium Rhizome), and 3.0 g of the root of* Angelica acutiloba* (Japanese Angelica Root). Kamishoyosan (Jiaweixiaoyaosan) (daily dose for human) consists of 3.0 g of the root of* Bupleurum falcatum* (Bupleurum Root), 3.0 g of Paeony root, 3.0 of Atractylodes Lancea Rhizome, 3.0 g of Japanese Angelica Root, 3.0 g of Poria Sclerotium, 2.0 g of the fruit of* Gardenia jasminoides* (Gardenia Fruit), 2.0 g of the root bark of* Paeonia suffruticosa* (Moutan Bark), 1.5 g of the root and stolon, with (unpeeled) or without (peeled) the periderm, of* Glycyrrhiza uralensis* (Glycyrrhiza), 1.0 g of the rhizome, with (unpeeled) or without (peeled) the periderm, of* Zingiber officinale* (Ginger), and 1.0 g of the terrestrial part of* Mentha arvensis* var.* piperascens* (Mentha Herb). Keishibukuryogan (Guizhifulingwan) (daily dose for human) consists of 3.0 g of the bark of the trunk of* Cinnamomum cassia* (Cinnamon Bark), 3.0 g of Paeony root, 3.0 g of the seed of Prunus persica (Peach Kernel), 3.0 g of Poria Sclerotium, and 3.0 g of Moutan Bark. These crude drugs were purchased from Daikoshoyaku (Nagoya, Japan) and were standardized by Japanese Pharmacopoeia 17th Edition [17]. The quality managers of the distributer identified and certificated the plant species, and the voucher specimens are deposited in Department of Pharmacognosy, Graduate School of Pharmaceutical Sciences, Nagoya City University, Nagoya, Japan. The mixtures of the above crude drugs' formula or each 10 g of single crude drug were boiled in 20 times the weight of water for 30 min and filtered. The decoction was lyophilized to yield powdered extract. Lot number and the extract ratio yielded are shown in Table 1. These extracts were suspended in distilled water to prepare the stock solution at a concentration of 0.1 g/ml and kept in −20°C until use.

Original dried extracts of tokishakuyakusan (TJ-23, Lot# 2150023010), kamishoyosan (TJ-24, Lot# 2160024010), and keishibukuryogan (TJ-25, Lot# 2150025010), that were also standardized by Japanese Pharmacopoeia 17th Edition [17], without any excipient as ethical Kampo extract formulation, were produced by and supplied from Tsumura Co., Ltd. (Tokyo).

Fingerprint patterns of these formulas were shown in Supplementary Figures 1–6. Each extract (25 mg) was suspended with MeOH (1 ml) and sonicated for 30 min. The supernatant (25 *μ*l) was injected to HPLC with the following conditions: system, Shimadzu LC–10A_VP_ (Kyoto, Japan); column, TSK–GEL ODS–80_TS_ (4.6 × 250 mm, Tosoh, Tokyo); mobile phase, 0.05 M AcOH–AcONH_4_ buffer (pH 3.6)/CH_3_CN 90:10 (0 min) – 0:100 (60 min), linear gradient; flow rate, 1.0 ml/min; column temperature, 40°C; and detection, 200 – 400 nm by a photodiode array detector. Some peaks were identified by the retention times and UV spectra of the standard compounds.

### 2.2. Materials

Pooled human liver microsomes, mixed gender (Lot# 610016), were bought from Sekisui XenoTech, LLC (Kansas City, KS, USA). Testosterone and* n*-butyl* p*-hydroxybenzoate (BHB) were bought from Nacalai Tesque (Kyoto, Japan). 6,7′-Dihydroxybergamottin (DHBG), *β*-NADPH, and 6*β*-hydroxytestosterone were purchased from ChemFaces (Wuhan, Hubei, China), Oriental Yeast (Tokyo, Japan), and Sigma Aldrich (St. Louis, MO, USA), respectively. The purities of DHBG and 6*β*-hydroxytestosterone were more than 98% according to the attached sheets.

### 2.3. Grapefruit Juice (GFJ)

A commercial GFJ (Lot#, 18.5.20 CK/3B, Toropicana 100, KIRIN Tropicana Inc., Tokyo, Japan) was purchased at a supermarket in Nagoya, Japan, in May, 2018. Ten ml of this GFJ was diluted with H_2_O and lyophilized to yield 1.0 g of dried powder. This powder was suspended in distilled water to prepare the stock solution at a concentration of 0.1 g/ml and kept in −20°C until use. Five-times diluted GFJ with MeOH was filtrated through 0.45 *μ*m filter, and then the content of DHBG was measured using HPLC as follows: system, Shimadzu LC–10A_VP_; column, Inertsil ODS-2 (4.6 × 250 mm, GL Science, Tokyo, Japan); mobile phase, A, 0.5% formic acid; B, acetonitrile containing 0.5% formic acid; A/B 20:80 (0 min), 50:50 (2 min), and 50:50 (15 min); flow rate, 1.0 ml/min; column temperature, 40°C; detection, absorbance at 310 nm; injection volume, 10 *μ*l; retention time of DHBG, 14.1 min. Linear regression of the concentration range of 0.2—2 *μ*g/ml of DHBG in methanol by the peak-area was calibrated with the least-squares method (*r*^2^=0.999), and the concentration of DHBG in the sample was calculated by this regression formula.

### 2.4. Assay for CYP3A4 Inhibition

We used slightly modified methods using rat liver microsome fraction as crude enzymes reported previously [9]. In brief, 10 *μ*l of the sample solution, 75 *μ*l of 0.15 M phosphate buffer (pH 7.4) containing 0.13 M EDTA, 1.0 *μ*l of 20 mM testosterone (dissolved in 20% MeOH), and 4 *μ*l of human liver microsome (3 mg/ml diluted with phosphate buffer) were mixed and incubated at 37°C for 5 min. Then, 10 *μ*l of 1 mM *β*-NADPH and 10 *μ*l of 6 mM MgCl_2_ were added, mixed, and incubated at 37°C for 15 min. The reaction was stopped by the addition of 100 *μ*l of 2-propanol containing 0.1 M BHB as an internal standard into the reactant and mixed vigorously. After centrifugation (12,000 ×* g*, 5 min), 25 *μ*l of the supernatant was applied to the following HPLC analysis using the same system except for mobile phase H_2_O/MeOH 40:60 and detection absorbance at 238 nm. Retention times of testosterone and BHB were 23.0 min, 7.9 min, and BHB, respectively. The linear regression of the concentration range of 0.013—0.20 mM of 6*β*-hydroxytestosterone by the peak-area ratio of 6*β*-hydroxytestosterone to BHB was calibrated with the least-squares method (*r*^2^=0.999). Data were expressed as relative activity (%) that was the ratio of the content of 6*β*-testosterone incubated with the sample to that incubated without the sample. The assay was conducted in triplicate, and the mean ± SD was exhibited. The half-maximum inhibitory concentration (IC_50_) was calculated from the least square regression line made from 3 points that crossed 50% of the control logarithmic concentration values.

### 2.5. Statistics

Statistical analysis was carried out by one-way analysis of variance and Dunnett's multiple comparison* t*-test using PASW Statistics software (version 18, SPSS; IBM, Armonk, NY, USA). A probability value of less than 0.05 was considered statistically significant.

## 3. Results

### 3.1. Inhibitory Effect of Grapefruit Juice on CYP3A4

**Figure 1:**
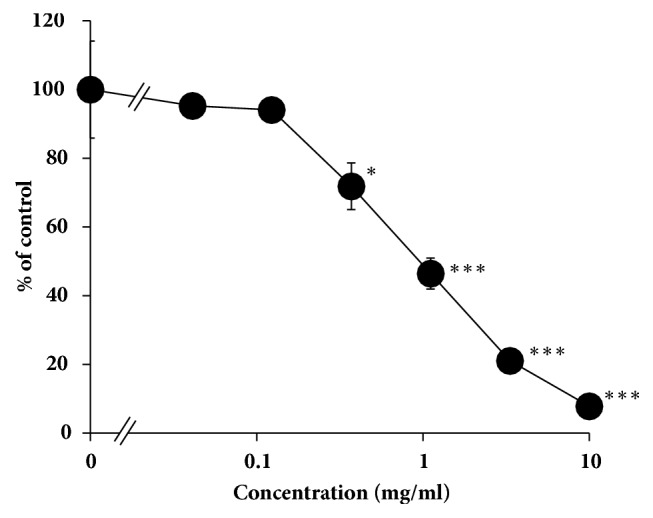
Inhibitory effect of the grapefruit juice (GFJ) used in this study on CYP3A4. Data are expressed as the mean ± SD (*n* = 3).

The grapefruit juice used in the present study contained 3.1 *μ*g/ml. This grapefruit juice inhibited CYP3A4 in dose-dependent manners with IC_50_ value of 0.96 mg/ml (Figure 1). This value is related to 1:104 diluted solution of the original grapefruit juice.

### 3.2. Inhibitory Effects of Tokishakuyakusan, Kamishoyosan, and Keishibukuryogan Extracts on CYP3A4

**Figure 2:**
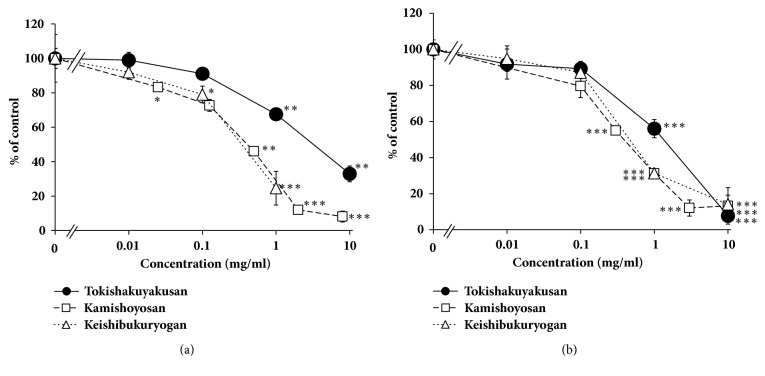
Inhibitory effect of tokishakuyakusan, kamishoyosan, and keishibukuryogan extracts on CYP3A4. (a) Each extract of Kampo formula was prepared from crude drug mixture described in Materials and Methods. (b) Each extract of Kampo formula was original dried extract of ethical Kampo extract formulation supplied from Tsumura Co., Ltd. Data are expressed as the mean ± SD (*n* = 3).

The extracts of tokishakuyakusan, kamishoyosan, and keishibukuryogan prepared in our laboratory or supplied by pharmaceutical industry inhibited CYP3A4 in dose-dependent manners (Figure 2). IC_50_ values of the extracts were shown in Table 1.

### 3.3. Inhibitory Effects of the Extracts of Crude Drugs Composing of Tokishakuyakusan, Kamishoyosan, and Keishibukuryogan on CYP3A4

**Figure 3:**
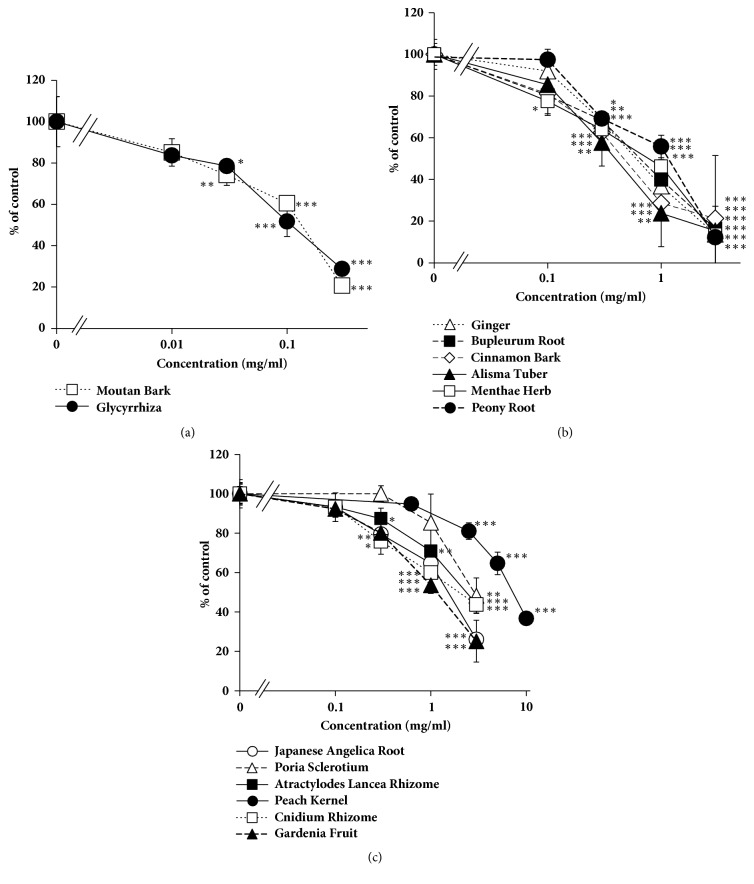
Inhibitory effect of the extracts of crude drugs composing tokishakuyakusan, kamishoyosan, and keishibukuryogan extracts on CYP3A4. Data are expressed as the mean ± SD (*n* = 3).

The extracts of crude drugs composing of tokishakuyakusan, kamishoyosan, and keishibukuryogan prepared in our laboratory inhibited CYP3A4 in dose-dependent manners (Figure 3). IC_50_ values of the extracts were shown in Table 1.

## 4. Discussion

In the present study, the extract of tokishakuyakusan, kamishoyosan, and keishibukuryogan, frequently prescribed Kampo formula in gynecological clinics, inhibited CYP3A4* in vitro*, with IC_50_ value of 3.1, 0.38, and 0.25 mg/ml. The one-time dosage of the extracts of tokishakuyakusan, kamishoyosan, and keishibukuryogan (3 times a day, t.i.d.) is 2.8, 2.4, and 0.82 g, and when patients take the extract with a cup of water (200 ml), the concentrations of the extracts are calculated as 14, 12, and 4.1 mg/ml, respectively. These concentrations are 4.5, 32, and 16-times higher than their IC_50_ values, and these ratios seem large to cause the inhibition* in vivo*. However, since the results of CYP inhibitions in* in vitro* experiments tended to exhibit excessive high potency [14],* in vitro* results should be treated carefully.

In this study, the original concentration of GFJ used was 100 mg/ml, and its IC_50_ value on CYP3A4 was 0.96 mg/ml. Since GFJ is the only natural substance to inhibit CYP3A4 in clinical studies [14], these values can be used as references to discuss the titer of inhibitory effect on CYP3A4 in clinics in comparison with* in vitro* inhibitory effects. The ratio of IC_50_ of GFJ to the concentration of original GFJ is calculated as 1:104; this value can be used as the coefficient for the extrapolation from* in vitro* results in the clinical situations. Ratio of inhibitory titer of the sample to GFJ is defined as the following formula:

(1) Ratio  of  inhibitory  titer  to  GFJ=IC50  of  GFJConc.  of  GFJ×IC50  of  the  targetConc.  of  the  target

Using this formula, the inhibitory effect of tokishakuyakusan, kamishoyosan, and keishibukuryogan extracts on CYP3A4 in clinics can be calculated as about 4.4 %, 30 %, and 16 % to that of GFJ (Table 1). The ratio 30% might be possible in clinics when a patient takes about 3-fold amount of the prescription, and it is possible that excessive dosage of kamishoyosan might cause drug interaction with the substrate of CYP3A4 in clinics. On the other hand, since tokishakuyakusan extract needs 23-fold amount of the prescription to exhibit the inhibitory effect as same as GFJ, it is considered that the possibility of causing drug interaction in clinics might be low.

When we prepare decoction of Kampo formula, the variation of crude drugs from batch to batch must not be avoidable. In this study, we also evaluated the effect of ethical Kampo extract prescription of the same formula manufactured from pharmaceutical industry. Although the compositions and dosages of the formulas were the same, the extracting ratios of the products made in our laboratory were higher than those manufactured in the industry. This difference would be dependent on the scale of preparation; that is, the industry produced large scale of the extract at the same time though we prepared the extract of 1-day dosage in our laboratory. By this reason, IC_50_ value of keishibukuryogan extract prepared in the laboratory had lower than that of the extract supplied from the industry. However, although the extracting ratios of tokishakuyakusan and kamishoyosan prepared in the laboratory were larger than those supplied from the industry, IC_50_ values of the extract prepared in the laboratory were almost the same for kamishoyosan and larger for tokishakuyakusan compared with the extract from the industry. This may be caused by the differences of original crude drug batches used, and in order to provide drug information about Kampo medicine to clinics, we have to consider the variation of the products. Considering the inhibitory titers of both extracts, it is possible that excessive dosage of kamishoyosan may cause drug interaction with the substrate of CYP3A4 in clinics, but the possibilities of tokishakuyakusan and keishibukuryogan to cause drug interaction can be quite low. Indeed, in clinical study, when healthy females took ethical keishibukuryogan extract formulation for 7 days, it had no effect on CYP3A4 activity [18].

Among the herbal components of these three Kampo formulas, Moutan Bark and Glycyrrhiza had relatively high inhibitory effects on CYP3A4* in vitro*. Among 50 crude drugs used in traditional Chinese medicine, Moutan Bark extract exhibited the highest inhibitory effect on CYP3A4* in vitro* [19]. The inhibitory titer of Moutan Bark extract on CYP3A4 in clinics can be calculated as 21 % to that of GFJ; it is possible that Moutan Bark may cause drug interaction with the substrate of CYP3A4 in clinics when the patients take excessive dosage. There are several reports that Glycyrrhiza (licorice) extract or its main ingredient glycyrrhizin inhibits CYP3A4* in vitro*; its inhibitory titer was weak [20, 21]. This study exhibited that the inhibitory titer of Glycyrrhiza extract was calculated as 12 % to that of GFJ, and the possibilities of Glycyrrhiza to cause drug interaction with CYP3A4 substrate can be low.

In conclusion, this study provides the inhibitory titers of 3 Kampo formulas frequently used in gynecological clinics on CYP3A4 as quantitative forms and exhibited that these Kampo formulas would not cause drug interaction* via* CYP3A4 when the patients take regular dosage. Since it might be possible that kamishoyosan cause drug interaction* via* CYP3A4 in excessive dosage, clinical study of kamishoyosan might be demanded for the safe usage of Kampo medicines to prevent drug interaction.

## Figures and Tables

**Table 1:** The half-maximum inhibitory concentration (IC_50_) of the samples on CYP3A4 and the ratios of the titer to grapefruit juice.

Name of crude drug or formula	Lot number	Extracting ratio (%)	Extract (g) in one time dosage of crude drugs or Kampo formula^(a)^	Estimated concentration when taken^(a)^ (mg/ml)	IC_50_ (mg/ml)	Ratio of inhibitory titer to grapefruit juice (%)
Grapefruit juice	18.5.20 CK/3B	–	–	100	0.96	100

Moutan Bark	5I11M	43	0.43	2.2	0.10	21
Glycyrrhiza	6B22	27	0.27	1.3	0.11	12
Mentha Herb	2F18M	43	0.43	2.1	0.66	3.1
Peony Root	5D08M	41	0.41	2.1	0.81	2.4
Ginger	5J16M	22	0.22	1.1	0.62	1.7
Cinnamon Bark	5B23M	9.3	0.09	0.47	0.42	1.1
Bupleurum Root	3I05M	15	0.15	0.76	0.67	1.1
Gardenia Fruit	9I11M	25	0.25	1.3	1.1	1.1
Japanese Angelica Root	7B06M	41	0.41	2.1	1.9	1.0
Atractylodes Lancea Rhizome	5G13M	26	0.47	2.3	2.4	0.94
Alisma Tuber	6C07M	6.9	0.07	0.35	0.39	0.85
Cindium Rhizome	1F08M	47	0.26	1.3	2.0	0.63
Peach Kernel	3I18M	9.8	0.10	0.49	7.0	0.067
Poria Sclerotium	5H18M	3.1	0.03	0.16	3.0	0.050

Tokishakuyakusan	–	39	2.8	14	3.1	4.4
Kamishoyosan	–	32	2.4	12	0.38	30
Keishibukuryogan	–	17	0.82	4.1	0.25	16

Tokishakuyakusan (Tsumura)	2150023010	18	1.3	6.7	1.1	5.8
Kamishoyosan (Tsumura)	2160024010	18	1.3	6.7	0.40	16
Keishibukuryogan (Tsumura)	2150025010	12	0.58	2.9	0.72	3.9

(a) One-time dosages for crude drugs were set as 1 g. One time dosages for Kampo formula were set as one-third of daily dosage.

(b) Estimated concentration when one-time dosage extract was taken with 200 ml H_2_O.

## Data Availability

The data used to support the findings of this study are available from the corresponding author upon request.

## Supplementary Materials

Supplementary Materials Fingerprint patterns of the experimental materials prepared from crude drugs used in this study were shown in Supplementary Figures 1–6.
