# Supplementary material for: Inhibitory Effect of Japanese Traditional Kampo Formula Frequently Prescribed in Gynecological Clinics on CYP3A4
Source: Evid Based Complement Alternat Med. 2018 Oct 1;2018:4259603. doi: 10.1155/2018/4259603 (PMC6188721; doi:10.1155/2018/4259603)
Supplement: Supplementary Materials — Fingerprint patterns of the experimental materials prepared from crude drugs used in this study were shown in Supplementary Figures 1–6. [file 4259603.f1.pdf]

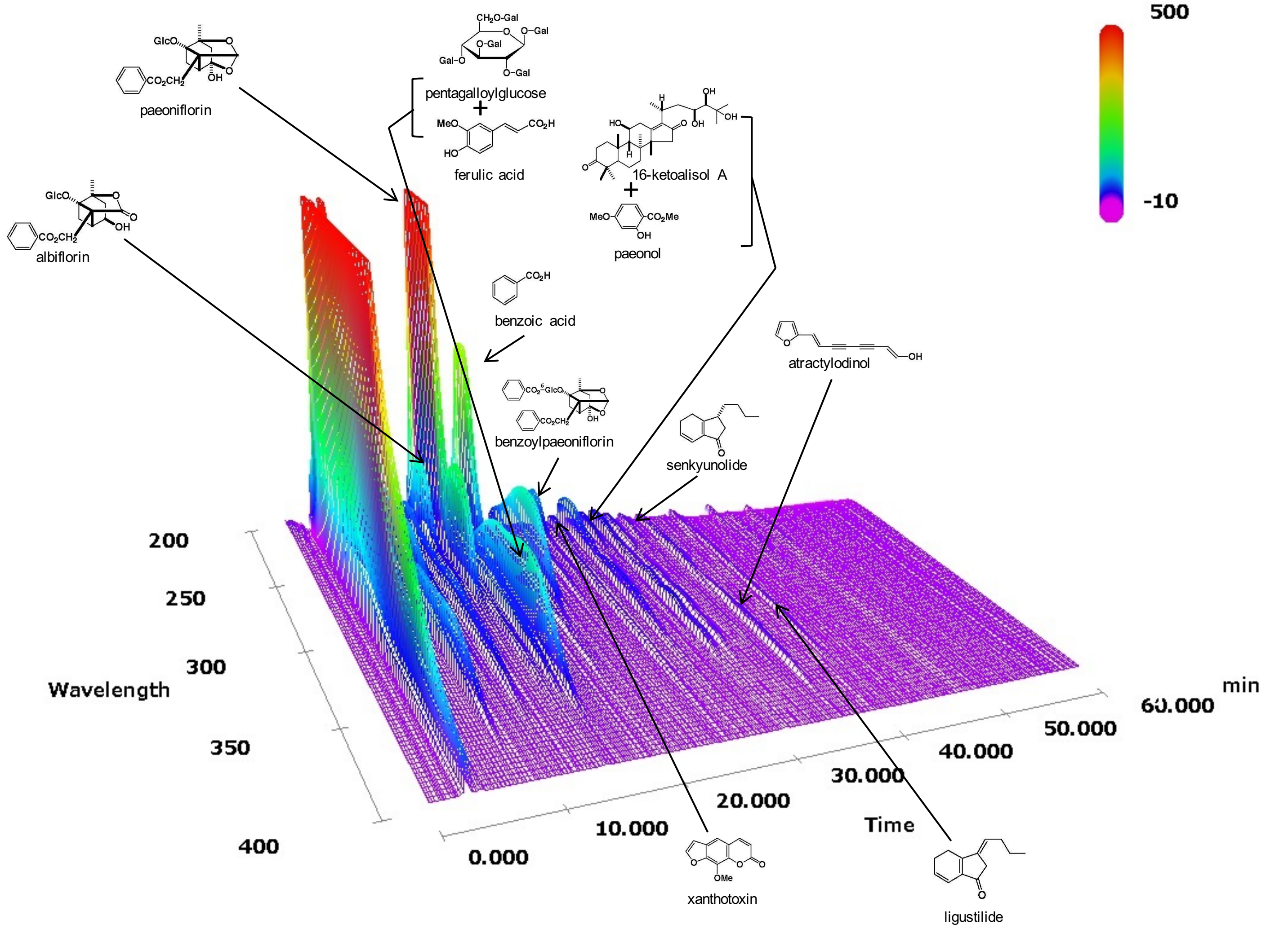

Supplemental Fig. 1
The extract of tokishakuyakusan prepared from crude drug mixture (25 mg) was suspended with MeOH (1 ml) and sonicated for 30 min. The supernatant (25 μl) was injected to HPLC with the following conditions: system, Shimadzu LC–10A$_{VP}$ (Kyoto, Japan); column, TSK–GEL ODS–80$_{TS}$ (4.6 × 250 mm, Tosoh, Tokyo); mobile phase, 0.05 M AcOH–AcONH$_4$ buffer (pH 3.6)/CH$_3$CN 90:10 (0 min) – 0:100 (60 min), linear gradient; flow rate, 1.0 ml/min; column temperature, 40°C; and detection, 200 – 400 nm by a photodiode array detector. Some peaks were identified by the retention times and UV spectra of the standard compounds.

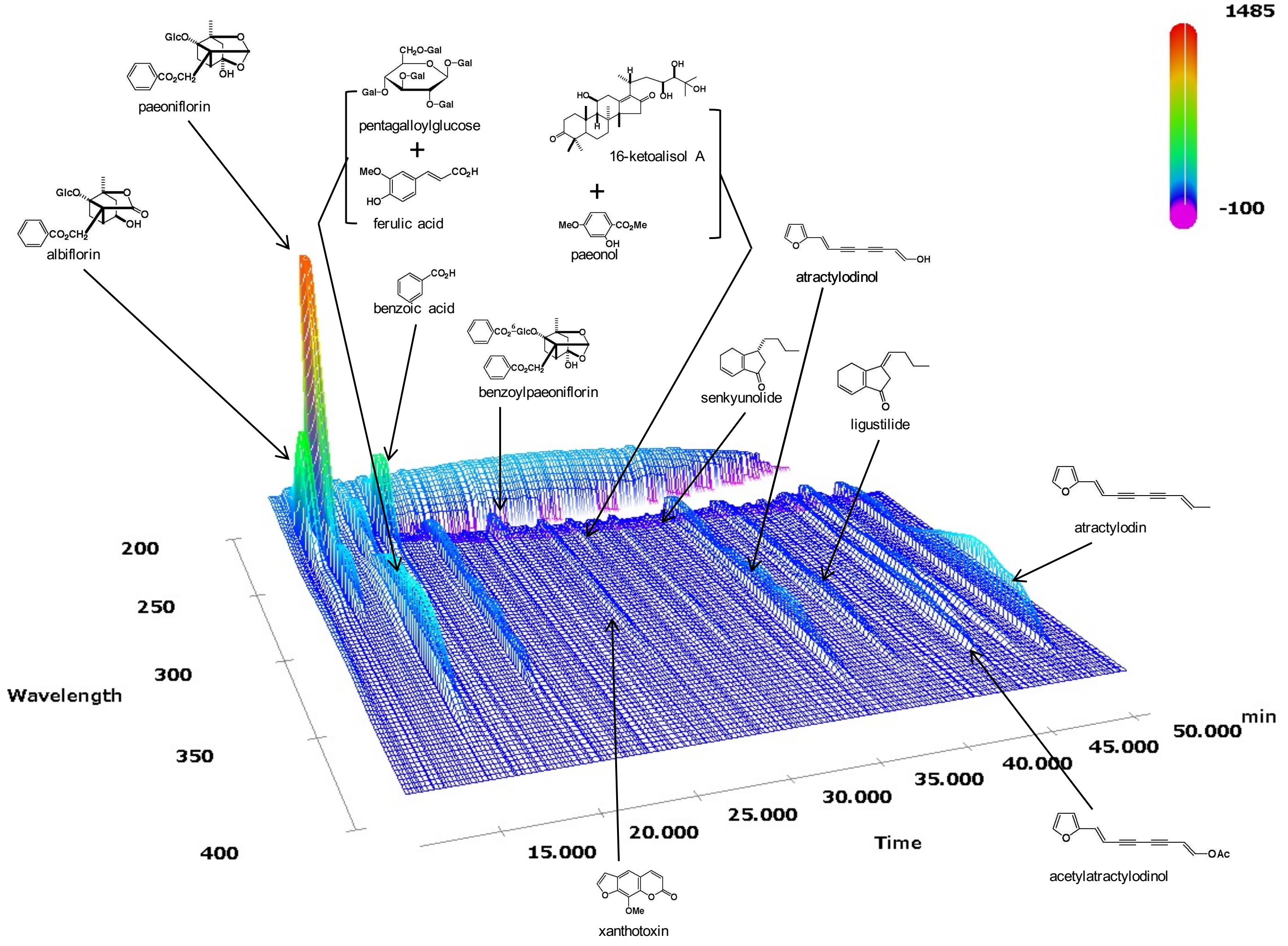

Supplemental Fig. 2
The extract of tokishakuyakusan supplied from Tsumura Co., Ltd. (25 mg) was suspended with MeOH (1 ml) and sonicated for 30 min. The supernatant (25 μl) was injected to HPLC with the following conditions: system, Shimadzu LC–10A$_{VP}$ (Kyoto, Japan); column, TSK–GEL ODS–80$_{TS}$ (4.6 × 250 mm, Tosoh, Tokyo); mobile phase, 0.05 M AcOH–AcONH$_4$ buffer (pH 3.6)/CH$_3$CN 90:10 (0 min) – 0:100 (60 min), linear gradient; flow rate, 1.0 ml/min; column temperature, 40°C; and detection, 200 – 400 nm by a photodiode array detector. Some peaks were identified by the retention times and UV spectra of the standard compounds.

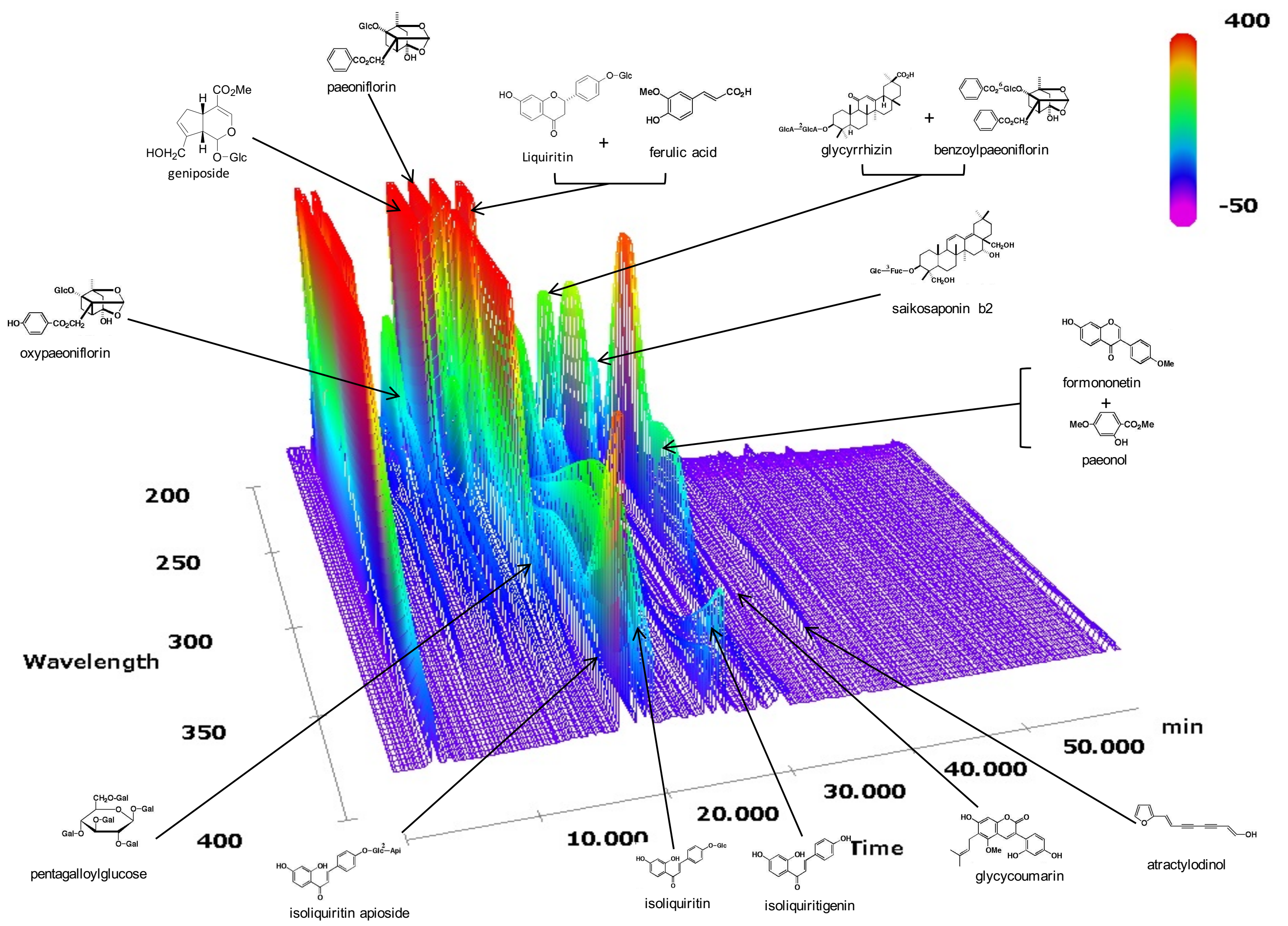

Supplemental Fig. 3

The extract of kamishoyosan prepared from crude drug mixture (25 mg) was suspended with MeOH (1 ml) and sonicated for 30 min. The supernatant (25 µl) was injected to HPLC with the following conditions: system, Shimadzu LC–10A$_{VP}$ (Kyoto, Japan); column, TSK–GEL ODS–80$_{TS}$ (4.6 × 250 mm, Tosoh, Tokyo); mobile phase, 0.05 M AcOH–AcONH$_4$ buffer (pH 3.6)/CH$_3$CN 90:10 (0 min) – 0:100 (60 min), linear gradient; flow rate, 1.0 ml/min; column temperature, 40°C; and detection, 200 – 400 nm by a photodiode array detector. Some peaks were identified by the retention times and UV spectra of the standard compounds.

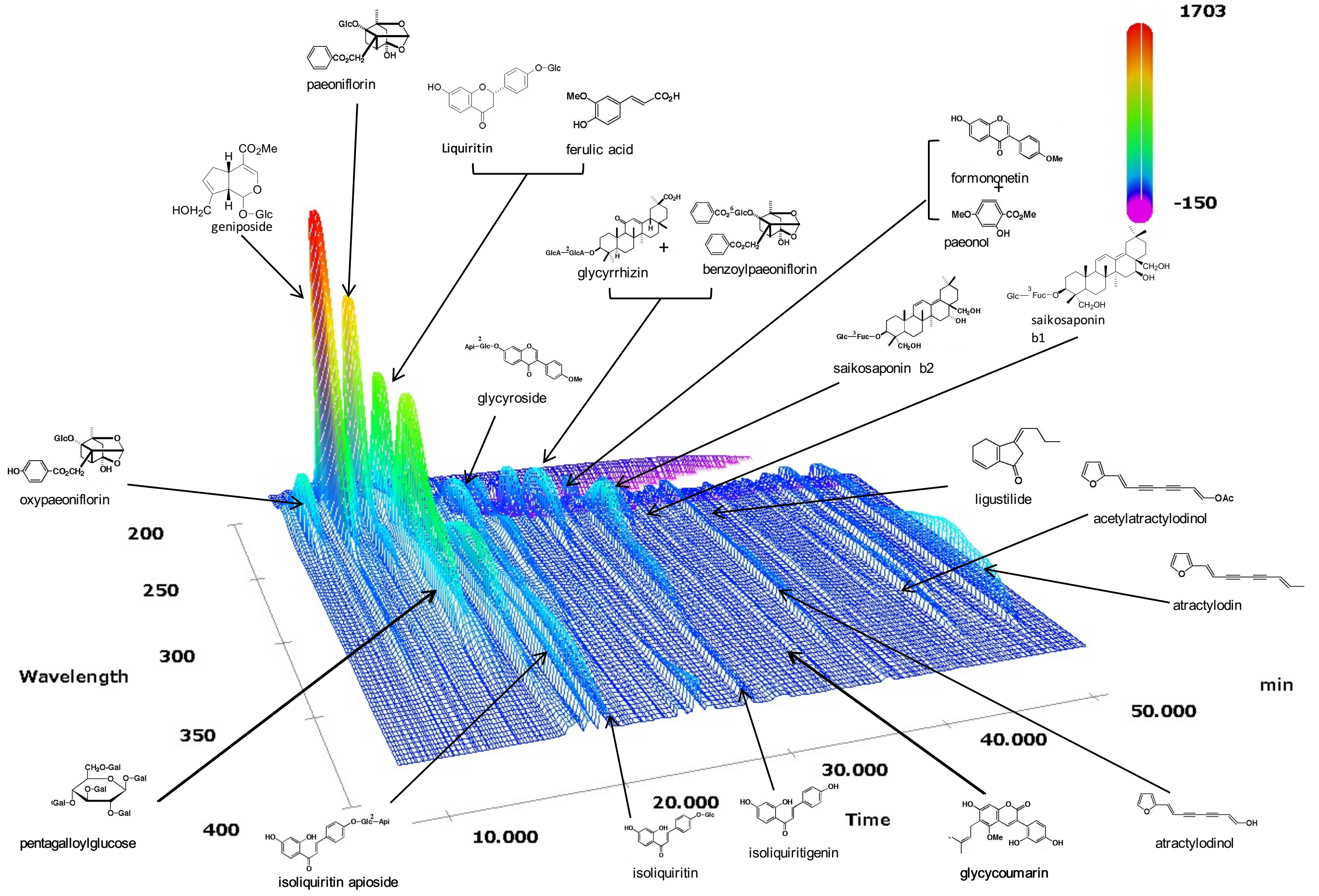

Supplemental Fig. 4
The extract of kamishoyosan supplied from Tsumura Co., Ltd. (25 mg) was suspended with MeOH (1 ml) and sonicated for 30 min. The supernatant (25 μl) was injected to HPLC with the following conditions: system, Shimadzu LC–10A$_{VP}$ (Kyoto, Japan); column, TSK–GEL ODS–80$_{TS}$ (4.6 × 250 mm, Tosoh, Tokyo); mobile phase, 0.05 M AcOH–AcONH$_4$ buffer (pH 3.6)/CH$_3$CN 90:10 (0 min) – 0:100 (60 min), linear gradient; flow rate, 1.0 ml/min; column temperature, 40°C; and detection, 200 – 400 nm by a photodiode array detector. Some peaks were identified by the retention times and UV spectra of the standard compounds.

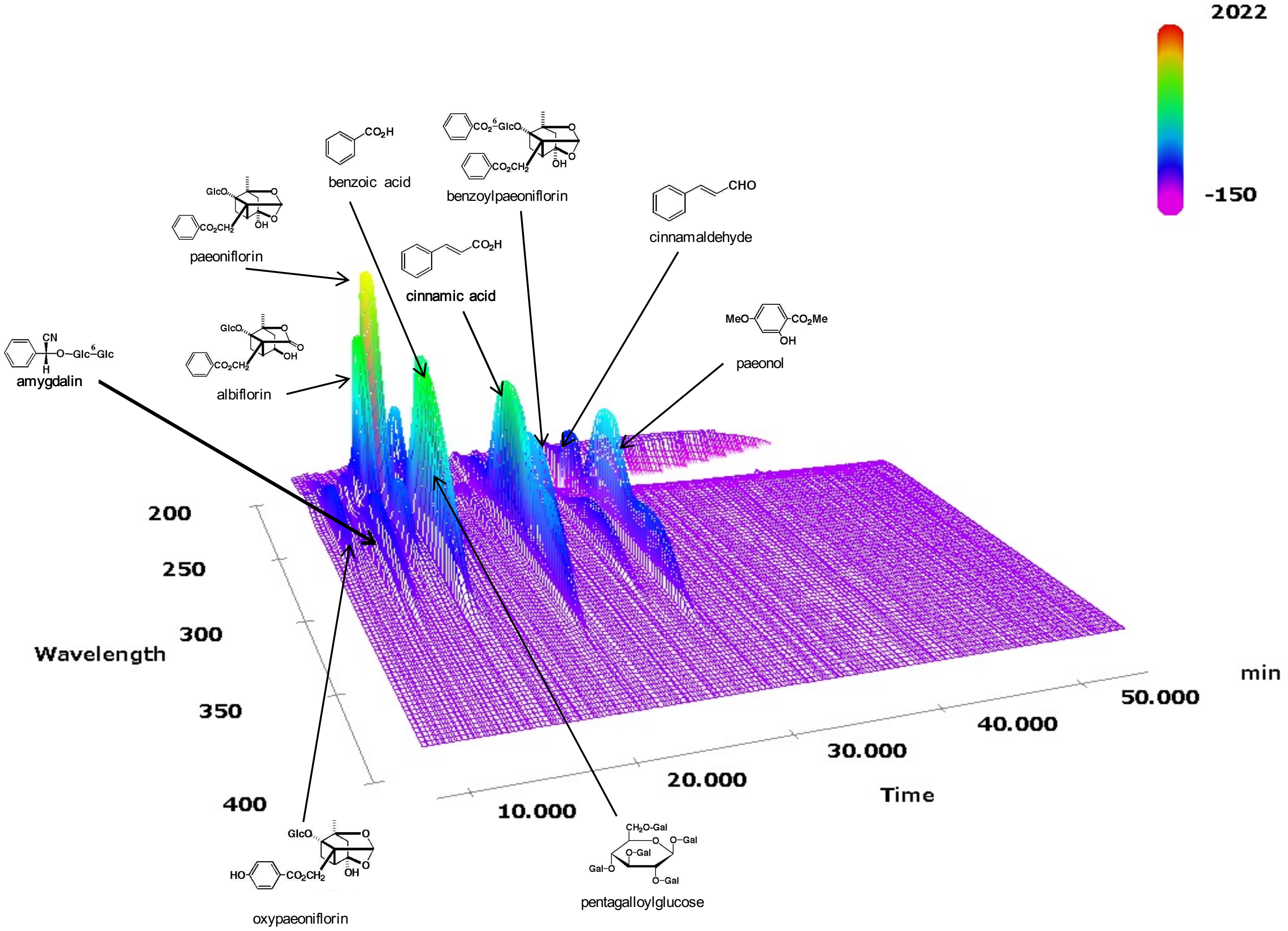

Supplemental Fig. 5
The extract of keishibukuryogan prepared from crude drug mixture (25 mg) was suspended with MeOH (1 ml) and sonicated for 30 min. The supernatant (25 µl) was injected to HPLC with the following conditions: system, Shimadzu LC–10A$_{VP}$ (Kyoto, Japan); column, TSK–GEL ODS–80$_{TS}$ (4.6 × 250 mm, Tosoh, Tokyo); mobile phase, 0.05 M AcOH–$AcONH_4$ buffer (pH 3.6)/CH_3CN 90:10 (0 min) – 0:100 (60 min), linear gradient; flow rate, 1.0 ml/min; column temperature, 40°C; and detection, 200 – 400 nm by a photodiode array detector. Some peaks were identified by the retention times and UV spectra of the standard compounds.

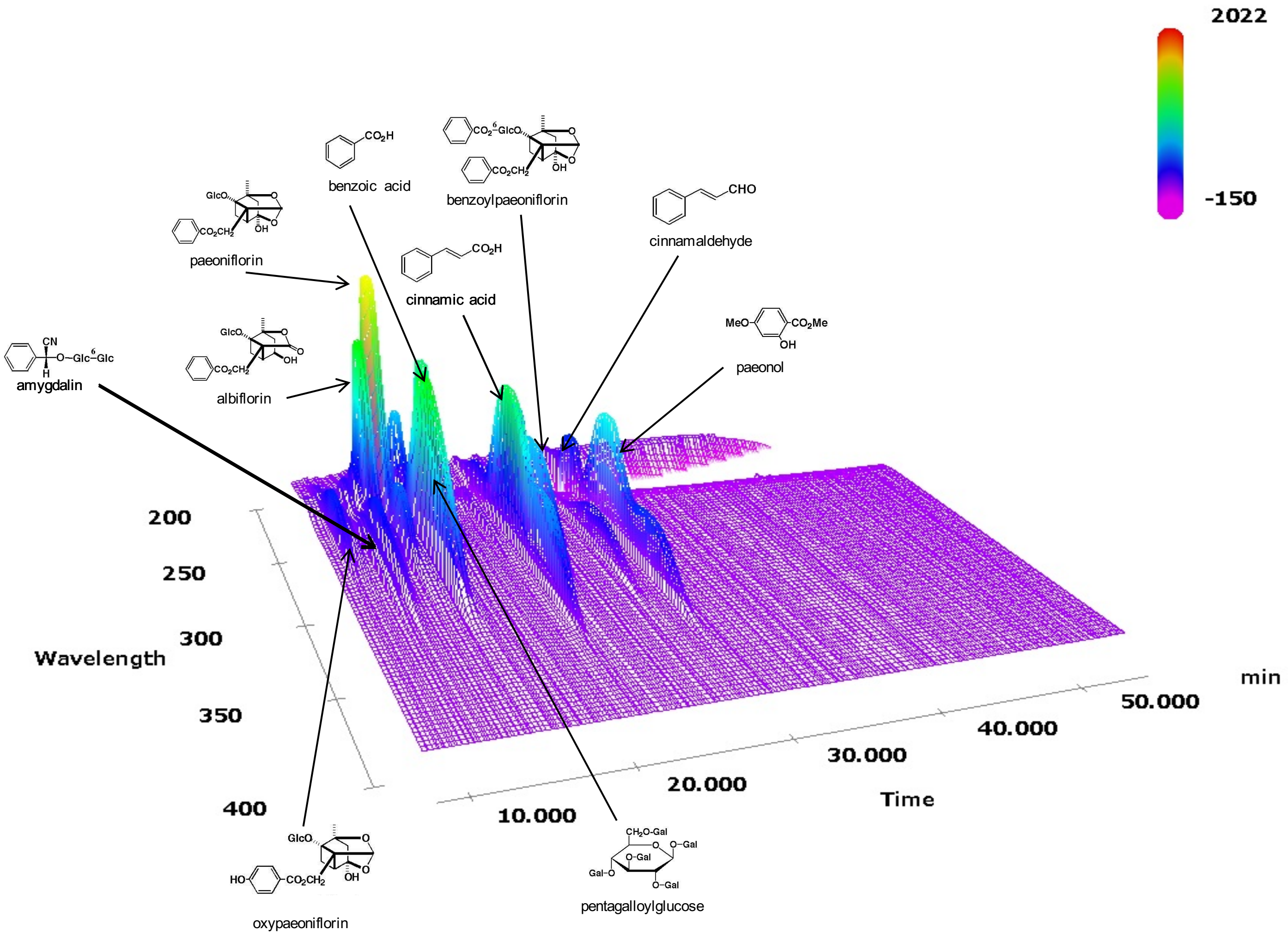

Supplemental Fig. 6

The extract of keishibukuryogan supplied from Tsumura Co., Ltd. (25 mg) was suspended with MeOH (1 ml) and sonicated for 30 min. The supernatant (25 µl) was injected to HPLC with the following conditions: system, Shimadzu LC–10A$_{VP}$ (Kyoto, Japan); column, TSK–GEL ODS–80$_{TS}$ (4.6 × 250 mm, Tosoh, Tokyo); mobile phase, 0.05 M AcOH–$AcONH_4$ buffer (pH 3.6)/CH_3CN 90:10 (0 min) – 0:100 (60 min), linear gradient; flow rate, 1.0 ml/min; column temperature, 40°C; and detection, 200 – 400 nm by a photodiode array detector. Some peaks were identified by the retention times and UV spectra of the standard compounds.
